# The Wider Impacts of the Coronavirus Pandemic on the NHS^*^

**DOI:** 10.1111/1475-5890.12227

**Published:** 2020-06-26

**Authors:** Carol Propper, George Stoye, Ben Zaranko

**Affiliations:** ^1^ Imperial College Business School; Institute for Fiscal Studies; ^2^ Institute for Fiscal Studies; University College London; ^3^ Institute for Fiscal Studies

**Keywords:** coronavirus, COVID‐19, treatment disruptions, National Health Service

## Abstract

The coronavirus pandemic has had huge impacts on the National Health Service (NHS). Patients suffering from the illness have placed unprecedented demands on acute care, particularly on intensive care units (ICUs). This has led to an effort to dramatically increase the resources available to NHS hospitals in treating these patients, involving reorganisation of hospital facilities, redeployment of existing staff and a drive to bring in recently retired and newly graduated staff to fight the pandemic. These increases in demand and changes to supply have had large knock‐on effects on the care provided to the wider population. This paper discusses likely implications for healthcare delivery in the short and medium term of the responses to the coronavirus pandemic, focusing primarily on the implications for non‐coronavirus patients. Patterns of past care suggest those most likely to be affected by these disruptions will be older individuals and those living in more deprived areas, potentially exacerbating pre‐existing health inequalities. Effects are likely to persist into the longer run, with particular challenges around recruitment and ongoing staff shortages.

## I. Introduction

The coronavirus pandemic has had huge impacts on the National Health Service (NHS). Patients suffering from the illness have placed unprecedented demands on acute care, particularly on intensive care units (ICUs). This has led to an effort to dramatically increase the resources available to NHS hospitals in treating these patients, involving reorganisation of hospital facilities, redeployment of existing staff and a drive to bring in recently retired and newly graduated staff to fight the pandemic.

These increases in demand and changes to supply have not only affected patients with the coronavirus, but also had large knock‐on effects on the care provided to the wider population. In this paper, we discuss likely implications for healthcare delivery in the short and medium term of the responses to the coronavirus pandemic, focusing primarily on the implications for non‐coronavirus patients. We first provide some context by briefly describing some of the increasing pressures felt in the NHS over recent years (Section II). We then set out the amount of planned and unplanned hospital care that we might have expected to take place in the absence of the pandemic and describe how this activity varies across age, socio‐economic status and place (Section III). In Section IV, we discuss how the amount and quality of care could be affected. To do this, we draw primarily on past data on NHS activity in England to understand who is most likely to be affected by these changes and on evidence from previous research on the impacts of sudden changes in the demand for, and supply of, healthcare. Our analysis, though speculative due to lack of real‐time data, suggests that disruptions to non‐coronavirus care are likely to have most impact on older and less affluent individuals. In Section V, we consider two longer‐term concerns: the impacts on staffing issues and on waiting times for elective care.

## II. Dealing with coronavirus patients against a backdrop of shortage

The immediate impact of the coronavirus pandemic on the NHS has been a huge increase in the demand for acute care and, in particular, intensive care facilities. The existing evidence for the UK strongly suggests that the virus affects some groups of the population more severely than others: men, older individuals, and those from Asian and black ethnic groups are at increased risk of death from COVID‐19.^1^ In response to the demand for care from coronavirus patients, there have been concerted efforts to boost hospital capacity – most notably in the creation of seven NHS Nightingale Hospitals – but this is against a backdrop of relatively few spare ICU beds and existing staff shortages.

Historically, the number of ICU beds in the UK has been low by international standards.^2^ The number of beds has increased in recent years, but occupancy remains high, with 83 per cent of the 4,123 adult critical care beds in England occupied in January 2020.^3^ The UK also has low levels of staff compared with other EU countries. For example, in 2015, the UK had fewer doctors for its population size than other European countries, at 2.8 doctors per 1,000 people compared with an EU15 average of 3.9.^4^ While the total number of doctors and nurses has increased in the last decade, this has been outstripped by the increase in activity. Between 2010 and 2016, the number of full‐time‐equivalent (FTE) doctors rose by just under 10 per cent, and nurses by around 1 per cent, compared with increases of around 17 per cent in the number of inpatient admissions and almost 25 per cent in the number of outpatient appointments.^5^ Nursing vacancies were almost 44,000 in England in the first quarter of 2019–20, a figure equivalent to around 12 per cent of the nursing workforce.^6^

## III. The effect on care volumes: cancellations, delays and disrupted treatment

To deal with coronavirus cases, the amount of resources available for non‐coronavirus patients has been reduced, though there are no real‐time publicly available data available to be able to precisely quantify by how much and for whom.

NHS England announced in March its intention to free up around 30,000 of its 100,000 general and acute hospital beds.^7^ Around half of this was to be achieved through urgent discharge of patients who are medically fit to leave. The remaining half was to come through the postponement of all non‐urgent elective operations. This included procedures such as cataract surgery, joint replacements and gall‐bladder removal. In the first three months of 2018, in the midst of a worse‐than‐usual flu season, 25,502 elective operations were cancelled in England.^8^ The scale of the current pandemic means that the eventual number of cancelled or delayed procedures is likely to be considerably higher this time around, with the number of affected patients potentially running into the hundreds of thousands.

In addition to hospitals delaying or cancelling treatment, some patients will have opted to postpone or decided against seeking treatment in an attempt to avoid visiting a hospital (potentially storing up health problems for the future). There is clear evidence of this in emergency care:^9^ the number of accident and emergency (A&E) attendances fell by almost half in the final weeks of March. This included particularly sharp reductions in the number of younger patients (aged between 15 and 44) presenting with asthma‐like symptoms and in the number of children attending with bronchitis or bronchiolitis. Some of this is possibly due to self‐isolation and/or the decrease in traffic, pollution and workplace accidents following the fall in economic activity. Some will be due to patients trying to avoid visiting the hospital. There are signs that A&E attendances have started to rebound in May, and the number of attendances for some ailments – such as gastrointestinal and cardiac conditions – has risen to close to or above the seasonally expected level. This is not true across the board, however, and total A&E attendances remain considerably lower than we would expect for this time of year. Nonetheless, emergency admissions usually make up around half of admissions to hospitals and there will be ongoing requirements for (non‐coronavirus‐related) emergency care.

We can use data on past NHS activity in England to examine who is likely to be affected by changes to their care, either due to cancellations or delays to elective treatment or due to reductions in emergency care. The immediate effects of the pandemic are currently projected to be highest between April and June this year. We use data from hospital records that cover this same period from a past year (2017) to examine which groups are most likely to be affected by potential disruptions to elective and non‐coronavirus emergency care. If disruptions persist for longer than this three‐month period then the affected number of patients will be even larger.

Between April and June 2017, a total of 1.7 million patients were admitted for 2.5 million elective procedures (both ‘urgent’ and ‘non‐urgent’) across England. In addition, 1.2 million patients were admitted to NHS hospitals a total of 1.5 million times for emergency (unplanned) reasons over the same period. And while these latter numbers may fall this year through reduced attendances at A&E, a large number of emergency cases will still need to be treated.

**FIGURE 1:**
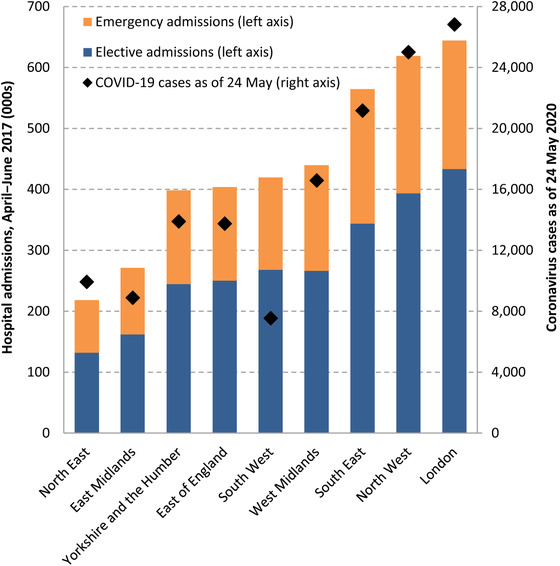
Total number of NHS elective and emergency admissions between April and June 2017, and coronavirus cases as of 24 May 2020, by English region *Source*: Authors’ calculations using Hospital Episode Statistics 2017 and HM Government (2020).

Figure 1 shows there is considerable regional variation in both the level of admissions and the split between emergency and elective procedures. As the NHS delays or cancels non‐urgent elective procedures, regions in which elective care represents a greater share of hospital activity (London, the North West and the South West) may be better placed to substitute resources towards tackling the coronavirus, although the extent to which elective resources can be used to treat emergency patients effectively remains uncertain.

The effect of the coronavirus outbreak to date has also differed across regions. At the time of writing, the number of cases in London far exceeds the number in any other region. Regions will therefore have differed both in how many coronavirus cases they have had to deal with and in their capacity to redeploy existing resources towards treating those patients.

**FIGURE 2:**
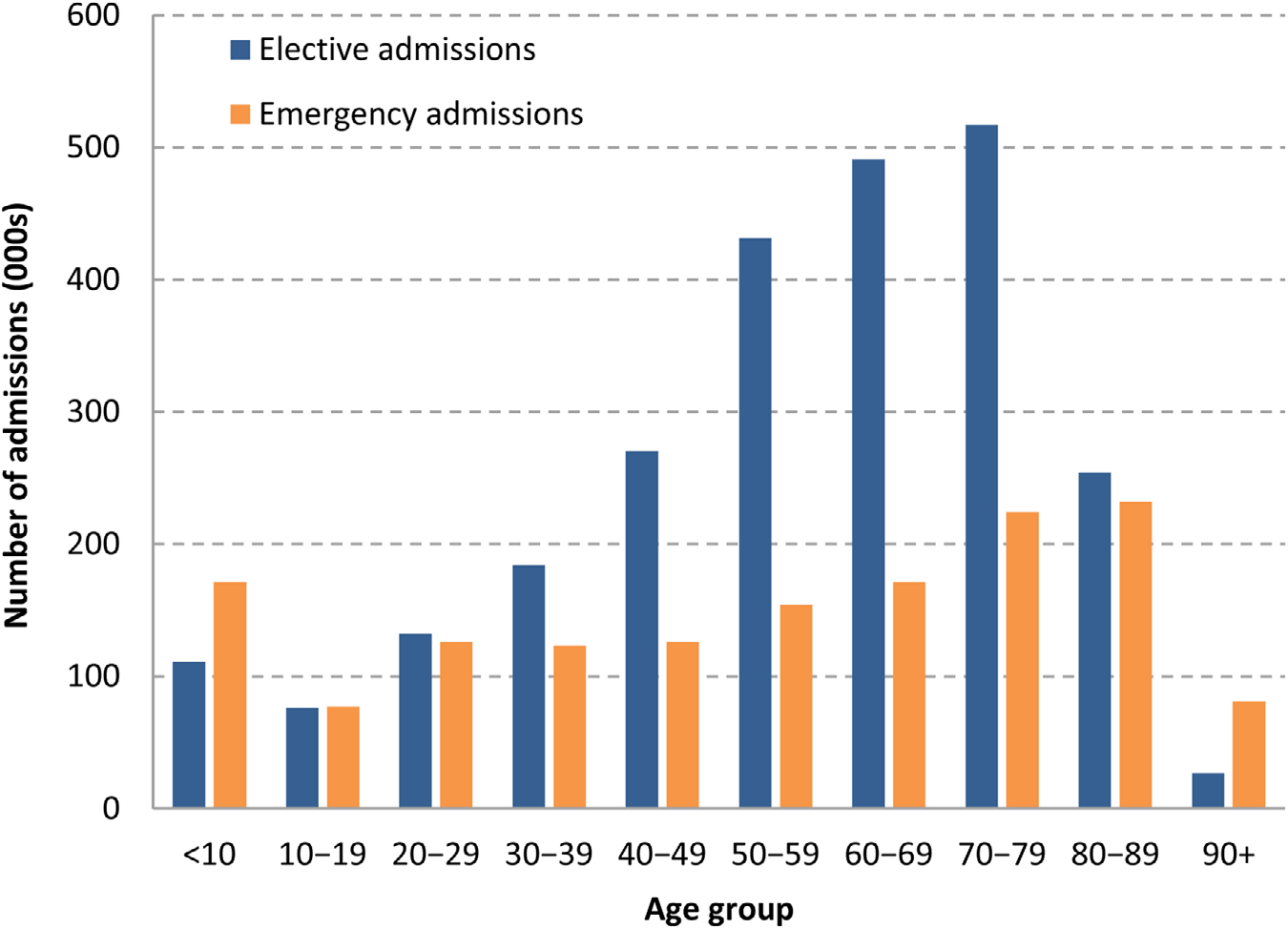
Number of elective and emergency admissions between April and June 2017, by ten‐year age group *Note*: Elective patients are those with pre‐planned admissions. Emergency patients are others, excluding maternity and delivery admissions. *Source*: Authors’ calculations using the Hospital Episode Statistics (HES) Admitted Patient Care data set. Includes all NHS‐funded patients treated in England between 1 April and 30 June 2017.

**FIGURE 3:**
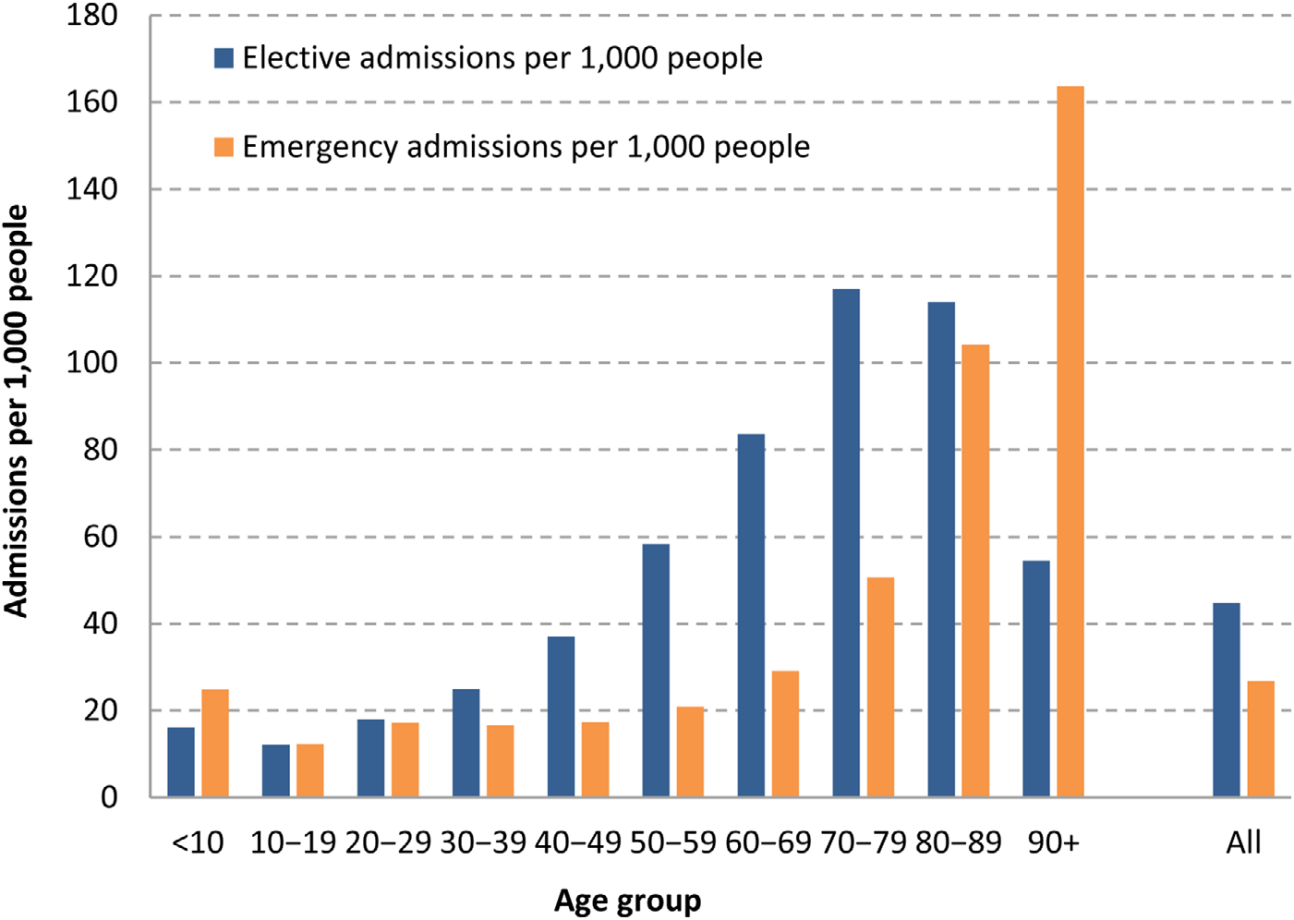
Rates of elective and emergency admissions between April and June 2017, by ten‐year age group *Note*: Elective patients are those with pre‐planned admissions. Emergency patients are others, excluding maternity and delivery admissions. *Source*: Authors’ calculations using the Hospital Episode Statistics (HES) Admitted Patient Care data set and Office for National Statistics (ONS) population estimates. Includes all NHS‐funded patients treated in England between 1 April and 30 June 2017.

It is also clear that the knock‐on effects of increased demand for coronavirus care will have had larger impacts on some age groups than others. Figure 2 shows the age distribution of NHS hospital admissions in England for April–June 2017. Emergency admissions are highest among children under the age of 10 (with 171,000 admissions over a three‐month period) and among those in their 70s and 80s. This pattern in part reflects the number of people in each age group. Figure 3 instead shows the number of admissions over the same April–June period per 1,000 people. The rate of emergency admissions is highest among the population aged 90 and above: 164 emergency admissions for every 1,000 people, versus 27 for the population as a whole. The very oldest in the population are therefore the most likely to have emergency care disrupted over the course of the pandemic.

Older people are also heavier users of elective hospital care than younger people. There were 517,000 elective admissions for people in their 70s compared with only 184,000 admissions for people in their 30s, despite the fact that there are 3 million more people in the younger age group living in England. Put another way, there were 117 and 114 elective admissions per 1,000 people for those in their 70s and 80s, respectively, but just 25 for every 1,000 people in their 30s. This means that the burden of delays to elective care is also most likely to have fallen on the older population (although rates of use of elective care are lower for the very oldest individuals aged 90 and above).

**FIGURE 4:**
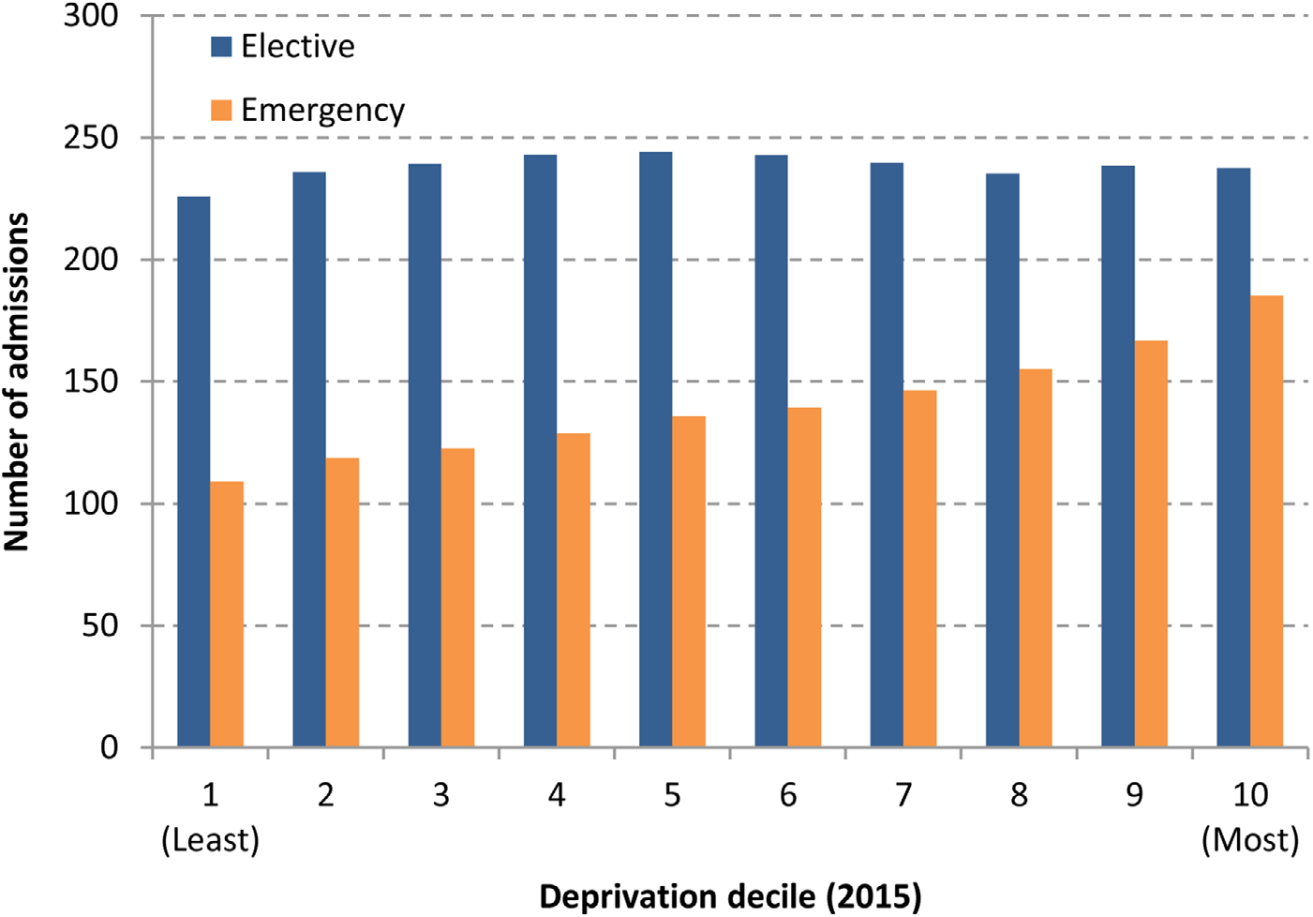
Elective and emergency admissions between April and June 2017, by local area deprivation *Note*: Elective patients are those with pre‐planned admissions. Emergency patients are others, excluding maternity and delivery admissions. Deprivation increases with decile number (10 = most deprived 10 per cent of areas). *Source*: Authors’ calculations using the Hospital Episode Statistics (HES) Admitted Patient Care data set. Includes all NHS‐funded patients treated in England between 1 April and 30 June 2017. Deprivation deciles defined using the ONS 2015 Index of Multiple Deprivation series.

It is also likely that the impact will have fallen unevenly across individuals of different socio‐economic status. Figure 4 shows the number of elective and emergency NHS hospital admissions in England between April and June 2017 across areas ranked by deprivation (as measured by the deprivation of the small area (LSOA) where the patient lives). Approximately a tenth of the population live in each decile, so the figures can also be interpreted as rates.

Elective admissions are relatively evenly distributed across patients from differently deprived areas, with between 225,000 and 250,000 admissions in each group during the period. By contrast, the number of emergency admissions is clearly higher among more deprived areas than in less deprived areas. In particular, there were 185,194 emergency admissions among residents living in the most deprived 10 per cent of areas of the country over the three‐month period, in comparison with only 108,948 in the least deprived 10 per cent of areas. Taken together, this suggests that disruptions to emergency care will have disproportionately affected older people and those who are the least affluent.

## IV. The effect on care quality

There have been huge increases in staff either coming out of retirement or joining the workforce early (for example, the Royal College of Nursing (RCN) estimated that 7,000 recently retired nurses returned in March,^10^ and final‐year nursing students have been doing clinical placements, adding perhaps another 3,000 nurses). Despite this, there will have been fewer staff available for treating other patients as more staff have focused on treating coronavirus patients. This is before accounting for the effects of the virus itself on staff numbers, with a March estimate from the Royal College of Physicians and the RCN suggesting that up to a quarter of NHS doctors and nurses had already had to take time off because of sickness or self‐isolation.^11^ Furthermore, staff are likely to have been working in unfamiliar settings. This raises the question of what is likely to happen to the outcomes of non‐coronavirus patients if they are treated by fewer, or by different, staff than usual.

We know from existing work that the reduced availability of hospital staff – due to either illness or being reassigned elsewhere – is likely to have negative effects on quality of care and on patient outcomes. Gruber and Kleiner (2012) show that reductions in the numbers of nursing staff present in hospitals during nursing strikes in the US led to an increase in mortality and readmissions among patients, even after adjusting for changes in demographics and case mix in these hospitals. Needleman et al. (2011) show that hospital shifts with nurse staffing levels below the target level are associated with increased levels of mortality. Friedrich and Hackmann (2017) show that a reduction in nursing employment in Denmark had detrimental effects on the quality of care provided by hospitals.

Even if numbers have been temporarily shored up, the composition and experience of the team, as well as its size, matter for the care that is provided to patients. Bartel et al. (2014) show, in a US context, that when nursing teams were disrupted by the departure of an experienced member or the arrival of a new hire, team productivity fell and patient outcomes suffered as a result. Chan (2020) shows that experience matters for doctor decision‐making and resultant patient outcomes.

It is difficult to draw firm conclusions about the likely scale of the impact on patient outcomes from coronavirus‐related staffing disruptions, in part because the scale of those disruptions is not yet clear. But Gruber and Kleiner (2012), for example, show that in‐hospital mortality was almost 20 per cent higher for patients admitted during a nursing strike (the typical length of which was 19 days). The coronavirus outbreak will have likely caused similar levels of disruption to nursing teams, given that nurses displaying symptoms were unable to work for an extended period. Doctors and other healthcare workers have been similarly affected. We would therefore expect similar, and quite possibly even larger, adverse effects on patient outcomes.

In sum, given that NHS care is focused upon older and more deprived individuals, it is likely that those who will have borne the highest costs of the shift of resources away from care other than that for COVID‐19 are this population, and this will likely persist into the medium term. This is only likely to exacerbate existing inequalities in health across place and socio‐economic status.

## V. Longer‐term concerns

Finally, we consider two longer‐term impacts, both of which have implications for the public purse. First, it is clear that staffing issues will loom large. While staff have come out of retirement to deal with the immediate crisis, after it is over they will presumably return to retirement. In addition, concerns over the lack of personal protective equipment (PPE) may diminish the relative attractiveness of working in the NHS in the future. Britain's high death toll and well‐publicised lack of PPE may well affect its ability to recruit from overseas, and this is in addition to the pressures associated with leaving the European Union. The Conservatives’ 2019 general election manifesto already pledged to find an additional 50,000 nurses by 2024–25. The impact of the coronavirus may mean they need to find more. With many areas already experiencing nursing shortages, it is hard to see how this can be achieved without an increase in nurses’ wages in shortage areas. This in turn would push up the cost of the NHS for taxpayers.

Second, waiting times for elective care will rise. Whilst waiting times are still well below their historic levels in the early 1990s and before, they have been rising recently, causing public concern. The response to the coronavirus will only increase the length of waits. Historically, increased waiting times in the NHS have led to an increased demand for private care.^12^ While incomes and employment are currently hard hit by the social isolation measures that have been imposed, these measures are not likely to affect the incomes of all equally. For example, Joyce and Xu (2020) show that low earners are seven times as likely as high earners to have worked in a sector that is now shut down. Some better‐off individuals may respond to rising waiting times and staff shortages by purchasing private care. This increased demand may lead in turn to more staff who currently work in the NHS choosing to work in the private sector. If this arises, it will exacerbate existing health inequalities.

On the other hand, during the current pandemic, there has been a groundswell in support for the NHS, and satisfaction with the health service had already risen recently, prior to the coronavirus pandemic.^13^ In the coming years, this may translate into greater support for tax rises to fund higher levels of health spending. Whatever the outcome, it is clear that the current crisis will mean that choices about how much to fund the NHS and how to spend those funds will be an even more central part of the political agenda.

## Footnotes

1 Williamson et al., 2020.

2 Rhodes et al., 2012.

3 NHS England, 2020a.

4 Lee and Stoye, 2018.

5 Lee, Propper and Stoye, 2020.

6 Gershlick and Charlesworth, 2019.

7
https://www.england.nhs.uk/coronavirus/wp-content/uploads/sites/52/2020/03/20200317-NHS-COVID-letter-FINAL.pdf.

8 NHS England, 2020b.

9 Public Health England, 2020.

10
https://www.rcn.org.uk/news-and-events/news/uk-thousands-return-to-nursing-to-help-during-covid-19-pandemic-230320.

11
https://news.sky.com/story/coronavirus-one-in-four-nhs-doctors-sick-or-in-isolation-11965886.

12 Besley, Hall and Preston, 1999.

13 Appleby et al., 2020.
